# 3,3-Dimethyl-2-benzofuran-1(3*H*)-one

**DOI:** 10.1107/S1600536811052913

**Published:** 2011-12-14

**Authors:** M. S. Siddegowda, Ray J. Butcher, Sema Ozturk Yıldırım, Mehmet Akkurt, H. S. Yathirajan, A. R. Ramesha

**Affiliations:** a Department of Studies in Chemistry, University of Mysore, Manasagangotri, Mysore 570 006, India; b Department of Chemistry, Howard University, 525 College Street NW, Washington, DC 20059, USA; c Department of Physics, Faculty of Sciences, Erciyes University, 38039 Kayseri, Turkey; d R. L. Fine Chem, Bengaluru 560 064, India

## Abstract

In the title compound, C_10_H_10_O_2_, all the non-H atoms except the methyl C atoms lie on a crystallographic mirror plane. In the crystal, C—H⋯O hydrogen bonds link the mol­ecules into zigzag chains running parallel to [100]. Weak π–π stacking inter­actions between the benzene rings [centroid–centroid distance = 3.9817 (5) Å] link the chains in the [010] direction.

## Related literature

For related structures, see: Fun *et al.* (2010 ▶, 2011 ▶).
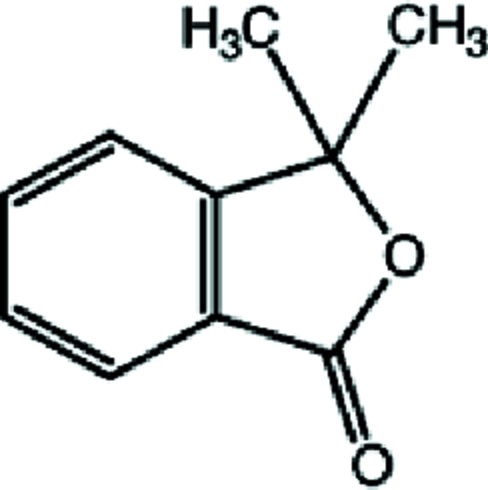


## Experimental

### 

#### Crystal data

C_10_H_10_O_2_
*M* *_r_* = 162.18
Orthorhombic, 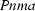

*a* = 14.3537 (9) Å
*b* = 7.0069 (5) Å
*c* = 8.2605 (5) Å
*V* = 830.80 (9) Å^3^
*Z* = 4
Mo *K*α radiation
μ = 0.09 mm^−1^
*T* = 123 K
0.55 × 0.44 × 0.30 mm

#### Data collection

Oxford Diffraction Xcalibur Ruby Gemini diffractometer
Absorption correction: refined from Δ*F* (*XABS2*; Parkin *et al.*, 1995 ▶) *T*_min_ = 0.952, *T*_max_ = 0.974
1840 measured reflections
1840 independent reflections
1454 reflections with *I* > 2σ(*I*)

#### Refinement

*R*[*F* ^2^ > 2σ(*F* ^2^)] = 0.052
*wR*(*F* ^2^) = 0.133
*S* = 1.09
1840 reflections
71 parameters
H-atom parameters constrained
Δρ_max_ = 0.34 e Å^−3^
Δρ_min_ = −0.30 e Å^−3^

### 

Data collection: *CrysAlis PRO* (Oxford Diffraction, 2007 ▶); cell refinement: *CrysAlis PRO*; data reduction: *CrysAlis RED* (Oxford Diffraction, 2007 ▶); program(s) used to solve structure: *SHELXS97* (Sheldrick, 2008 ▶); program(s) used to refine structure: *SHELXL97* (Sheldrick, 2008 ▶); molecular graphics: *ORTEP-3* (Farrugia, 1997 ▶); software used to prepare material for publication: *WinGX* (Farrugia, 1999 ▶) and *PLATON* (Spek, 2009 ▶).

## Supplementary Material

Crystal structure: contains datablock(s) global, I. DOI: 10.1107/S1600536811052913/hb6560sup1.cif

Structure factors: contains datablock(s) I. DOI: 10.1107/S1600536811052913/hb6560Isup2.hkl

Supplementary material file. DOI: 10.1107/S1600536811052913/hb6560Isup3.cml

Additional supplementary materials:  crystallographic information; 3D view; checkCIF report

## Figures and Tables

**Table 1:** Hydrogen-bond geometry (Å, °)

*D*—H⋯*A*	*D*—H	H⋯*A*	*D*⋯*A*	*D*—H⋯*A*
C2—H2⋯O2^i^	0.93	2.43	3.3072 (17)	158

Symmetry code: (i) 
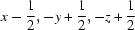
.

**Fig. 1.**
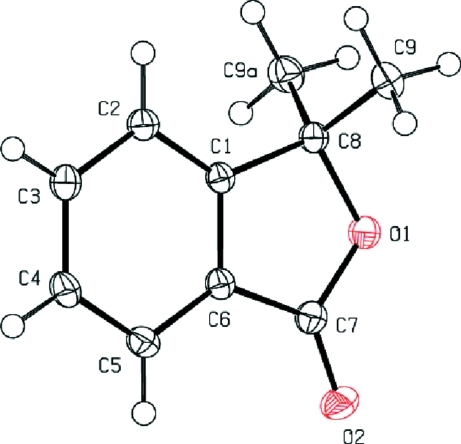
The title molecule (I) showing displacement ellipsoids for non-H atoms drawn at the 50% probability level.

**Fig. 2.**
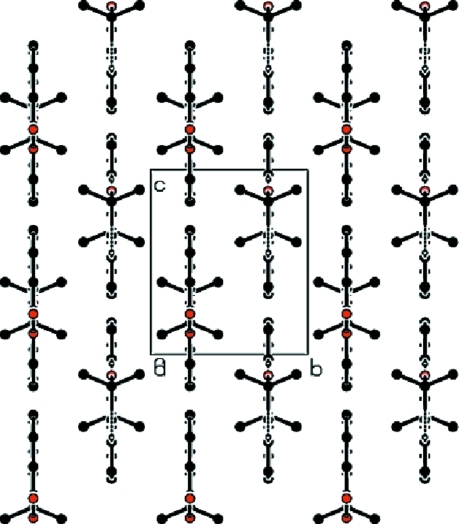
View of the packing and hydrogen bonding of (I) down a axis.

**Fig. 3.**
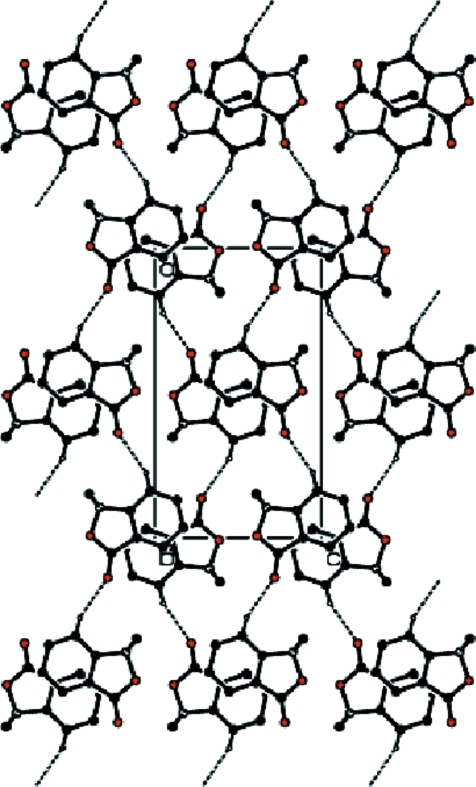
View of the packing and hydrogen bonding of (I) down b axis.

**Fig. 4.**
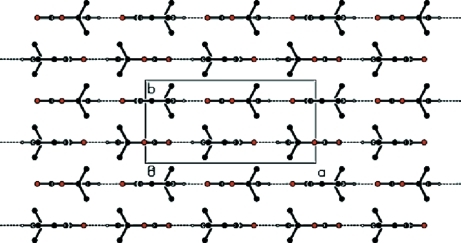
View of the packing and hydrogen bonding of (I) down c axis.

## supplementary crystallographic information

### Comment

3,3-Dimethylisobenzofuran-1(3*H*)-one, (I), is used to synthesize 10,10-dimethylanthrone (Fun *et al.*, 2010). It is a one of the intermediate for melitracenium chloride (Fun *et al.*, 2011). We now report its stucture.

In the title molecule (I), (Fig. 1), a mirror plane passes through the remaining atoms of the molecule, except the atoms of two methyl groups which are *mirror images of each other*.

In the crystal, intermolecular C—H ··· O hydrogen bonds link molecules forming zigzag chains in the layer parallel to the (101) plane and along the *a* axis (Table 1, Fig. 2a,b,c). Furthermore, there exist weak π-π stacking interactions [*Cg*2···*Cg*2(2 - *x*, -1/2 + *y*, -*z*) = 3.9817 (5) Å, *Cg*2···*Cg*2(2 - *x*, 1/2 + *y*, -*z*) = 3.9817 (5) Å, *Cg*2···*Cg*2(2 - *x*, -*y*, -*z*) = 3.9817 (5) Å, *Cg*2···*Cg*2(2 - *x*, 1 - *y*, -*z*) = 3.9817 (5) Å] between the C1–C6 benzene rings along the [010] direction.

### Experimental

The title compound was obtained as a gift sample from *R. L*. Fine Chemicals, Bengaluru, India.  Colourless prisms of (I) were grown from toluene solution by slow evaporation (m.p.: 337–340 K).

### Refinement

All H atoms were added in calculated positions and refined as riding with C–H distances of 0.93 and 0.96 Å. The isotropic atomic displacement parameters of H atoms were fixed to 1.2 or 1.5 ×*U*_eq_ of the parent atom.

### Crystal data

**Table:**

C_10_H_10_O_2_	*F*(000) = 344
*M**_r_* = 162.18	*D*_x_ = 1.297 Mg m^−^^3^
Orthorhombic, *P**n**m**a*	Mo *K*α radiation, λ = 0.71073 Å
Hall symbol: -P 2ac 2n	Cell parameters from 1444 reflections
*a* = 14.3537 (9) Å	θ = 2.8–35.2°
*b* = 7.0069 (5) Å	µ = 0.09 mm^−^^1^
*c* = 8.2605 (5) Å	*T* = 123 K
*V* = 830.80 (9) Å^3^	Prism, colourless
*Z* = 4	0.55 × 0.44 × 0.30 mm

### Data collection

**Table:**

Oxford Diffraction Xcalibur Ruby Gemini diffractometer	1840 independent reflections
Radiation source: Enhance (Mo) X-ray Source	1454 reflections with *I* > 2σ(*I*)
graphite	*R*_int_ = 0.000
Detector resolution: 10.5081 pixels mm^-1^	θ_max_ = 35.2°, θ_min_ = 2.8°
ω scans	*h* = 0→22
Absorption correction: part of the refinement model (Δ*F*) (*XABS2*; Parkin *et al.*, 1995)	*k* = 0→11
*T*_min_ = 0.952, *T*_max_ = 0.974	*l* = 0→13
1840 measured reflections	

### Refinement

**Table:**

Refinement on *F*^2^	Primary atom site location: structure-invariant direct methods
Least-squares matrix: full	Secondary atom site location: difference Fourier map
*R*[*F*^2^ > 2σ(*F*^2^)] = 0.052	Hydrogen site location: inferred from neighbouring sites
*wR*(*F*^2^) = 0.133	H-atom parameters constrained
*S* = 1.09	*w* = 1/[σ^2^(*F*_o_^2^) + (0.0591*P*)^2^ + 0.0987*P*] where *P* = (*F*_o_^2^ + 2*F*_c_^2^)/3
1840 reflections	(Δ/σ)_max_ < 0.001
71 parameters	Δρ_max_ = 0.34 e Å^−^^3^
0 restraints	Δρ_min_ = −0.30 e Å^−^^3^

### Special details

**Table:**

Experimental. Absorption correction: XABS2 (Parkin *et al.*, 1995); cubic fit to sinθ/λ - 24 parameters
Geometry. Bond distances, angles *etc*. have been calculated using the rounded fractional coordinates. All su's are estimated from the variances of the (full) variance-covariance matrix. The cell e.s.d.'s are taken into account in the estimation of distances, angles and torsion angles
Refinement. Refinement on *F*^2^ for ALL reflections except those flagged by the user for potential systematic errors. Weighted *R*-factors *wR* and all goodnesses of fit *S* are based on *F*^2^, conventional *R*-factors *R* are based on *F*, with *F* set to zero for negative *F*^2^. The observed criterion of *F*^2^ > σ(*F*^2^) is used only for calculating -*R*-factor-obs *etc*. and is not relevant to the choice of reflections for refinement. *R*-factors based on *F*^2^ are statistically about twice as large as those based on *F*, and *R*-factors based on ALL data will be even larger.

### Fractional atomic coordinates and isotropic or equivalent isotropic displacement parameters (Å^2^)

**Table:**

	*x*	*y*	*z*	*U*_iso_*/*U*_eq_	
O1	0.99217 (7)	0.25000	0.38584 (11)	0.0216 (3)	
O2	1.13652 (7)	0.25000	0.28045 (13)	0.0260 (3)	
C1	0.90567 (9)	0.25000	0.14603 (15)	0.0159 (3)	
C2	0.83829 (9)	0.25000	0.02623 (16)	0.0193 (3)	
C3	0.86910 (10)	0.25000	−0.13404 (16)	0.0211 (3)	
C4	0.96384 (10)	0.25000	−0.17273 (16)	0.0208 (3)	
C5	1.03071 (9)	0.25000	−0.05143 (16)	0.0192 (3)	
C6	0.99948 (9)	0.25000	0.10807 (15)	0.0165 (3)	
C7	1.05334 (9)	0.25000	0.25962 (15)	0.0189 (3)	
C8	0.89464 (9)	0.25000	0.32851 (15)	0.0175 (3)	
C9	0.84773 (7)	0.07067 (15)	0.39183 (12)	0.0229 (3)	
H2	0.77510	0.25000	0.05140	0.0230*	
H3	0.82540	0.25000	−0.21720	0.0250*	
H4	0.98220	0.25000	−0.28070	0.0250*	
H5	1.09400	0.25000	−0.07580	0.0230*	
H9A	0.88010	−0.03960	0.35190	0.0340*	
H9B	0.78420	0.06740	0.35560	0.0340*	
H9C	0.84920	0.07080	0.50800	0.0340*	

### Atomic displacement parameters (Å^2^)

**Table:**

	*U*^11^	*U*^22^	*U*^33^	*U*^12^	*U*^13^	*U*^23^
O1	0.0185 (4)	0.0326 (5)	0.0136 (4)	0.0000	−0.0019 (4)	0.0000
O2	0.0169 (4)	0.0368 (6)	0.0242 (5)	0.0000	−0.0042 (4)	0.0000
C1	0.0169 (5)	0.0170 (5)	0.0137 (5)	0.0000	0.0004 (4)	0.0000
C2	0.0176 (5)	0.0251 (6)	0.0153 (5)	0.0000	−0.0002 (5)	0.0000
C3	0.0220 (6)	0.0270 (6)	0.0143 (5)	0.0000	−0.0021 (5)	0.0000
C4	0.0246 (6)	0.0248 (6)	0.0131 (5)	0.0000	0.0025 (5)	0.0000
C5	0.0185 (5)	0.0213 (5)	0.0177 (5)	0.0000	0.0031 (5)	0.0000
C6	0.0176 (5)	0.0173 (5)	0.0147 (5)	0.0000	0.0003 (4)	0.0000
C7	0.0182 (6)	0.0214 (5)	0.0172 (5)	0.0000	−0.0011 (5)	0.0000
C8	0.0164 (5)	0.0232 (6)	0.0130 (5)	0.0000	−0.0007 (4)	0.0000
C9	0.0259 (5)	0.0244 (4)	0.0185 (4)	−0.0011 (4)	0.0028 (3)	0.0028 (3)

### Geometric parameters (Å, °)

**Table:**

O1—C7	1.3631 (16)	C6—C7	1.4714 (18)
O1—C8	1.4779 (16)	C8—C9	1.5185 (13)
O2—C7	1.2063 (16)	C8—C9^i^	1.5185 (13)
C1—C2	1.3837 (18)	C2—H2	0.9300
C1—C6	1.3826 (18)	C3—H3	0.9300
C1—C8	1.5157 (18)	C4—H4	0.9300
C2—C3	1.3958 (19)	C5—H5	0.9300
C3—C4	1.397 (2)	C9—H9A	0.9600
C4—C5	1.3875 (19)	C9—H9B	0.9600
C5—C6	1.3917 (18)	C9—H9C	0.9600
			
C7—O1—C8	111.41 (9)	C1—C8—C9	112.89 (7)
C2—C1—C6	121.23 (12)	C1—C8—C9^i^	112.89 (7)
C2—C1—C8	129.66 (12)	C9—C8—C9^i^	111.68 (10)
C6—C1—C8	109.11 (11)	C1—C2—H2	121.00
C1—C2—C3	117.19 (12)	C3—C2—H2	121.00
C2—C3—C4	121.70 (12)	C2—C3—H3	119.00
C3—C4—C5	120.54 (12)	C4—C3—H3	119.00
C4—C5—C6	117.44 (12)	C3—C4—H4	120.00
C1—C6—C5	121.90 (12)	C5—C4—H4	120.00
C1—C6—C7	108.59 (11)	C4—C5—H5	121.00
C5—C6—C7	129.51 (12)	C6—C5—H5	121.00
O1—C7—O2	121.90 (12)	C8—C9—H9A	109.00
O1—C7—C6	108.20 (11)	C8—C9—H9B	109.00
O2—C7—C6	129.90 (12)	C8—C9—H9C	110.00
O1—C8—C1	102.69 (10)	H9A—C9—H9B	109.00
O1—C8—C9	108.04 (7)	H9A—C9—H9C	109.00
O1—C8—C9^i^	108.04 (7)	H9B—C9—H9C	109.00
			
C7—O1—C8—C9	119.51 (7)	C8—C1—C6—C5	180.00
C8—O1—C7—O2	180.00	C2—C1—C8—C9	63.92 (8)
C8—O1—C7—C6	0.00	C1—C2—C3—C4	0.00
C7—O1—C8—C1	0.00	C2—C3—C4—C5	0.00
C8—C1—C2—C3	180.00	C3—C4—C5—C6	0.00
C2—C1—C6—C5	0.00	C4—C5—C6—C1	0.00
C2—C1—C6—C7	180.00	C4—C5—C6—C7	180.00
C6—C1—C2—C3	0.00	C1—C6—C7—O2	180.00
C6—C1—C8—O1	0.00	C5—C6—C7—O1	180.00
C6—C1—C8—C9	−116.08 (8)	C5—C6—C7—O2	0.00
C8—C1—C6—C7	0.00	C1—C6—C7—O1	0.00
C2—C1—C8—O1	180.00		

Symmetry codes: (i) *x*, −*y*+1/2, *z*.

### Hydrogen-bond geometry (Å, °)

**Table:**

*D*—H···*A*	*D*—H	H···*A*	*D*···*A*	*D*—H···*A*
C2—H2···O2^ii^	0.93	2.43	3.3072 (17)	158

Symmetry codes: (ii) *x*−1/2, −*y*+1/2, −*z*+1/2.
